# A multicentric, 2 × 2 factorial, randomised, open-label trial to evaluate the clinical effectiveness of structured physical activity training and cognitive behavioural therapy versus usual care in heart failure patients: a protocol for the PACT-HF trial

**DOI:** 10.12688/wellcomeopenres.18047.1

**Published:** 2022-08-12

**Authors:** Panniyammakal Jeemon, Salim Reethu, Sanjay Ganapathi, Lakshmipuram Rajappan Lakshmi Kanth, Eapen Punnoose, Jabir Abdullakutty, Shafeeq Mattumal, Johny Joseph, Stigi Joseph, Chitra Venkateswaran, Poornima Sunder, Abraham Samuel Babu, Sebastian Padickaparambil, Kandagathuparambil Rajan Neenumol, Susanna Chacko, Shamla Shajahan, Krishnaja Krishnankutty, Selma Devis, Rani Joseph, Bhagavathikandy Shemija, Sneha Anna John, Sivadasanpillai Harikrishnan

**Affiliations:** 1 ICMR-Centre for Advanced Research and Excellence in Heart Failure (CARE-HF) and Achutha Menon Centre for Health Science Studies, Sree Chitra Tirunal Institute for Medical Sciences and Technology, Trivandrum, India; 2 ICMR-Centre for Advanced Research and Excellence in Heart Failure (CARE-HF), Sree Chitra Thirunal Institute of Medical Sciences and Technology, Trivandrum, Kerala, 695011, India; 3 MOSC Medical College Hospital, Kolenchery, India; 4 Lisie Hospital, Ernakulam, India; 5 Aster MIMS, Calicut, India; 6 Caritas Hospital, Kottayam, India; 7 Little flower hospital and research centre, Angamali, India; 8 Believers Church Medical College Hospital, Thiruvalla, India; 9 Mehac Foundation, Kochi, India; 10 Department of Physiotherapy, Manipal College of Health Professions, Manipal Academy of Higher Education, Manipal, India; 11 Department of Clinical Psychology, Manipal College of Health Professions, Manipal Academy of Higher Education, Manipal, India

**Keywords:** Factorial trial, heart failure, cardiac rehabilitation, exercise, behaviour therapy, India

## Abstract

**Background:** Heart failure (HF) is a multi-morbid chronic condition, which adversely affects the quality of life of the affected individual. Engaging the patient and their caregivers in self-care is known to reduce mortality, rehospitalisation and improve quality of life among HF patients. The PACT-HF trial will answer whether clinical benefits in terms of mortality and hospitalisation outcomes can be demonstrated by using a pragmatic design to explore the specific effects of physical activity, and cognitive behavioural therapy in HF patients in India.

**Methods:** We will conduct a 2 × 2 factorial, randomized, open-label trial, which aims to see if rehabilitation strategies of structured physical activity training and cognitive behavioural therapy for depression and self-management reduce the risk of repeat hospitalisation and deaths in HF patients in India. Patients will be randomised to (1) physical activity + usual care (2) cognitive behaviour therapy + usual care, (3) physical activity + cognitive behaviour therapy + usual care, and (4) usual care at 1:1:1:1 ratio. Time to mortality will be the primary outcome. A composite of mortality and hospitalisation for HF will be the main secondary outcome. Additional secondary outcomes will include ‘days alive and out of hospital’, cumulative hospitalisation, quality of life, Minnesota Living with Heart Failure questionnaire score, depression score, six minutes walking distance, handgrip strength, and adherence to medicines and lifestyle.  The effects of intervention on the primary outcome will be estimated from Cox proportional hazard models. For the continuous secondary outcome variables, differences between randomised groups will be estimated from linear mixed models or generalised estimating equations (GEE) as appropriate.

**Discussion:** PACT-HF is designed to provide reliable evidence about the balance of benefits and risks conferred by physical activity and cognitive behavioural therapy-based cardiac rehabilitation for those with HF, irrespective of their initial disease severity.

## Introduction

Heart failure (HF) is a multi-morbid chronic condition, which adversely impacts the quality of life of the affected individual ^1, 2^. The disease burden attributable to HF in India is increasing and the estimated prevalence ranges from 1.3 million to 4.6 million, with an annual incidence of 0.5–1.8 million ^3^. Heart failure requires resource-intensive treatment strategies, which are not immediately available and accessible for most patients in India. The financial burden of HF to the patients and their care givers is huge, and represents 2–3% of the total cost of all medical conditions ^4^. The estimated cost of HF treatment, resulting from frequent hospital admissions, multiple-drug therapy, comorbidities and device treatments ^4^ in 2012, is approximately $1186 million in India ^4^. The HF mortality in India is even worse than common cancers with an estimated 40% surviving past five years ^5^.

Besides the resource-intensive medical and medical device therapies, engaging the patient and their caregivers in self-care is known to reduce mortality, rehospitalisation and improve quality of life among HF patients ^6, 7^. In general, HF patients need to engage in a number of self-care behaviours like adherence to multiple medications, living a healthy life style ( *e.g.* no smoking or drinking alcohol), monitoring symptoms, identifying and addressing warning signs or symptoms, managing depression or psycho-social distress and other co-morbidities ^1, 8^. The ability to participate in and adhere to self-care behaviours becomes more challenging to people as HF symptoms worsen. Decline in physical function and increased psycho-social distress-related symptoms are common in HF patients as the disease worsens ^8– 10^. It may further hinder the individual’s capacity for performing self-care and deteriorates the patient condition in a vicious cycle ^11^.

Physical activity is an important core component of cardiac rehabilitation (CR) ^12^. Timely introduction of structured and graded physical activity based on the severity of HF symptoms is an important component of cardiac rehabilitation (CR) and is associated with improvements in functional capacity, and quality of life ^13^. Studies focusing on CR in India are limited. Even though preliminary works suggesting the benefits of exercise-based CR on functional capacity and quality of life exist ^14^, a structured physical activity-based programme that is culturally adapted to the Indian settings has not been evaluated for its effectiveness in reducing hospitalisation and mortality of HF patients.

Modification of negative thoughts and behaviours to positive and healthy ones with the help of cognitive behavioural therapy is another strategy to improve self-care in HF patients with symptoms of depression or psycho-social distress ^15^. Although cognitive behavioural therapy is demonstrated as a useful strategy in HF patients, the studies were smaller in terms of participants numbers, and with shorter follow-up. Further, the effectiveness of a culturally adapted behavioural therapy in reducing hospitalisation and mortality has not been evaluated in well-designed randomised controlled trials ^16^ in Indian settings.

The PACT-HF trial will answer whether clinical benefits in terms of mortality and hospitalisation outcomes can be demonstrated in HF patients by using a pragmatic design to explore the specific effects of physical activity, and cognitive behavioural therapy in HF patients in India.

## Methods

The study protocol conforms to the CONSORT reporting guidelines ^17^ and other relevant guidelines ^18^.

### Ethical considerations

The study protocol was approved by the institutional ethical committee of SCTIMST (SCT/IEC/1313.9/AUGUST-2021) and all participating centres. The trial is registered in clinical trial registry of India (CTRI) with registration number CTRI/2020/11/028808. Written informed consent will be obtained from all study participants.

### Study design

We will conduct a 2×2 factorial, randomised, and open label trial to evaluate the specific effects of physical activity and behavioural therapy in reducing mortality and hospitalisation in HF patients in India. The patients recruited in the study will be randomly assigned to one of four intervention groups: a control arm (usual care-C), intervention A (physical activity), intervention B (cognitive behaviour therapy), and a combination of intervention A and B (physical activity + cognitive behaviour therapy) ^19^. We assumed no interaction between the treatment arms. 

### Study settings

The study will be carried out in six hospital settings with facilities for management of HF in Kerala, India. Medium to large tertiary care referral hospitals in both private and public settings will be selected for the study with representation from the Southern, Central and Northern parts of Kerala, India.

### Study population

Eligible patients with physician-diagnosed HF ^2^ will be invited from the selected hospitals for participation in the trial. HF patients with New York Heart Association (NYHA) class I to IV will be invited to participate in the study. Adults over 18 years old, citizens of India, permanent residents of the state of Kerala, who speak Malayalam language and with physician-diagnosed HF will be included in the trial. We will exclude bedridden patients, patients with known psychiatric disorders and under treatment, and those who refuse to provide written informed consent from the trial. Further, we will exclude currently enrolled patients in other formal cardiac rehabilitation programs.

### Screening and enrolment

All invited participants will be informed about the trial procedures and written informed consent will be obtained from each of them. A detailed participant information sheet will be made available in the local language and will be given to each participant. The recruitment of the patients in the study will be conducted from September 2021 to September 2022 in all study centres. All the identified and consenting participants will be screened based on inclusion and exclusion criteria by a dedicated research nurse ^20^. A structured study questionnaire will be used for documenting the screening data ^21^.

### Intervention and comparator

Intervention A (physical activity and usual care): Participants will be asked to perform physical activity, along with a structured exercise program of moderate intensity for at least 30 minutes, five days a week throughout the study period.

Intervention B (cognitive behaviour therapy and usual care): Participants will receive individual face-to-face behavioural therapy sessions once a month and telephone sessions once every week.

Intervention C (physical activity + cognitive behaviour therapy and usual care): Participants will receive both behaviour therapy intervention and exercise regime.

Comparator: Participants in the usual care arm will continue without any intervention as suggested above.

### Intervention delivery

A dedicated research nurse with experience in managing HF patients and clinical trials will be recruited in each centre to deliver the intervention. A TIDieR check list ^22^ will be circulated among investigators to describe the nature of the intervention and the role of the study team in delivering the intervention along with their expertise ( *Extended data*) ^23, 24^.

The research nurses identified in each centre will be given training on cognitive behaviour therapy and physical activity under the guidance of experts in the field. A detailed trainers’ manual and lesson plan will be used for training purposes. Training by experts in the field will be given in a full-day face-to-face session. This will be reinforced by online sessions for clarification of queries and through mock sessions. A compendium containing standard operating procedures, study proforma, reference manual and a logbook to record patient details and narratives will be provided and explained to the research nurse at each site.

The exercise regimen will be demonstrated to the patient in the HF clinic under the supervision of the treating cardiologist. Individually tailored, low-intensity exercises of short duration will be offered initially as a strategy to enhance adherence. The intensity of the exercise will be gradually increased based on tolerance as assessed by Borg’s rating of perceived exertion. The patients enrolled in the cognitive behaviour therapy arm will be given direct in-person as well as telephonic sessions over a period of one year. Upon enrolment, participants will receive a manual on behaviour therapy and physical activity and recorded videos of the exercise program.

### The intervention and training modules

***Resource module for physical activity*.** The resource module consists of three manuals: The trainer’s manual (English), and the patient’s manual in English and Malayalam versions (native language). The manual will be used to explain the intensity and duration of exercises with the help of visual art forms. A recorded video of exercises specially designed for HF patients with increasing intensity over time will be included as a tool of intervention. 

***Resource module for behaviour therapy*.** It consists of three manuals: The trainer’s manual (English), patient’s manual in English version, and patient’s manual Malayalam version (native language). A recorded video of breathing exercises (relaxation technique) for HF patients will be incorporated as part of the relaxation program. The manual will focus on the interaction between physical health and emotional health.

### Structured physical activity intervention

After the randomisation, the structured physical activity intervention group will receive face-to-face group training sessions by the research nurse in the outpatient settings of the respective study sites. All the intervention participants will be advised to participate in the group session. They will be asked to perform all their activities of daily living, regular physical activity ( *e.g.*, walking, gardening, and household work) along with their exercise of choice. Participants will be informed to engage in mild to moderate exercise for at least 30 minutes, five days a week. The exercise component will be demonstrated over 10 sessions and will include a warm-up and a cool-down session. The participants will be requested to increase the duration of the exercise session according to their tolerance. Yoga-based physical activity will be also introduced as part of the intervention. Very simple and specific yogasanas will be demonstrated to HF patients to increase their overall physical activity.

The participants will be instructed to perform each schedule of exercises and will be asked to increase the intensity gradually every month. The type and duration of exercises will change with the intensity of the exercise that they perform. All participants receiving the exercise training will be given a manual developed in Malayalam (native language) with all instructions. Further, a self-assessment checklist will be provided to capture the fidelity to the intervention regime ( *Extended data*) ^25^

Participation in the intervention session will be monitored using self-assessment forms. Participants will be asked to bring these self-assessment forms at each follow-up visit. A mobile phone application (containing the app Metronome Beats) will be used by patients to increase the intensity and duration of exercise. The research team will contact participants weekly through telephone and provide input on modifying and tailoring the exercise intensity and duration. 

### Cognitive behavioural therapy intervention

The cognitive behavioural therapy modules developed for the study will be simple, easy, and understandable such that the intervention can be given even by even a lay person. The behavioural therapy intervention will consist of individual sessions of 30–45 minutes duration, that will be delivered by a trained nurse. The sessions will be given in the outpatient settings of the study sites. We will use a detailed trainer’s manual to explain the sessions to the research nurse. The delivery of the brief behavioural counselling sessions will be based on a structured guidance document. Participants will receive individual face-to-face sessions once a month and telephone sessions once a week for the next 10 months. The cognitive behavioural therapy sessions will generally involve building rapport with patients, understanding patient’s thoughts and belief about HF, assessing their dysfunctional thoughts, teaching them methods to cope with the disease, incorporating positive thoughts, teaching them methods to relax the mind and reviewing the assignments given to each individual patient. At the end of each session, participants will be required to complete the ‘Mood thermometer ^26^’ to see how they feel each day and their mood changes throughout the therapy.

All participants receiving the cognitive behavioural therapy interventions will be given a behavioural therapy manual in their native language. This self-help manual will aid in guiding the participants in unsupervised home tasks and assignments based on behavioural therapy. A self-assessment checklist ( *Extended data*) ^27^ will be provided and it will help the participants in monitoring their progress and in attaining therapy-related goals during the follow-up periods.

### Outcome measures

Time to mortality will be the primary outcome for the proposed study. A composite outcome of mortality and hospitalisation for HF will be considered as the main secondary outcome. Additional secondary outcome variables will include ‘days alive and out of hospital (DAOH)’, cumulative hospitalisation rate, disease-specific and general quality of life, depression score, six minutes walking distance, handgrip strength, and adherence to medicines and lifestyle.

### Measurements

The socio-demographic details like age, gender, education, occupation will be recording during the patients’ first visit. Aetiology, history and risk factors for the disease will also be recorded at baseline. Furthermore, recent blood investigations done in the three months prior will be also analysed.

Participants will be seen every three months until the end of the three-year follow-up ^28^ will help to identify measurement details at different time points. At study visits, depression and anxiety scores will be collected from each participant by using the PHQ9 ^29^ and GAD-7 ^30^ questionnaires, respectively. The self- management component will include assessments of participant’s medication adherence, management of weight and diet, and family support as reported by the patient. The medication adherence will be measured using the four-item Morisky Green Levine medication adherence scale ^31^. We will use the multimorbidity treatment burden questionnaire (MTBQ) ^32^, to measure treatment burden. The study proforma will also assess the medical utilisation, patient satisfaction in quality of care, family support, and diet management.

We will use the Kansas City Cardiomyopathy Questionnaire (KCCQ) ^33^ and EQ-5D-5L ^34^ to assess quality of life. The KCCQ assess the disease specific quality of life. The EQ-5D-5L provides a simple descriptive profile and a single index value for health status. Standardized and validated instruments will be used for assessing the functional status, severity of disease, and treatment.

The six-minute walk test (6MWT) will be measured using standard settings during their out-patient visits ^35^. The 6MWT will be performed indoors, on a flat-surface corridor, which is 30 m long. Before starting the test, the research nurse will record the blood pressure, pulse rate, and oxygen saturation. The patient will be advised to start the test after being instructed on the test procedure. The test will run for six minutes and will follow all the instructions given by the American Thoracic Society ^36^. At the end of the test, the distance covered will be documented along with the pulse rate, blood pressure, oxygen saturation and Borg’s rating of perceived exertion and fatigue.

Hand grip strength will be assessed using standard assessment procedures using a dynamometer ^37^. In brief, hand dominance will be determined, following which the test will be carried out with the patient in a sitting position. The arm will be supported and positioned at right angles with the elbow. The forearm will be in the mid-prone position. The participant will be asked to make a tight fist without moving the wrist or the elbow while squeezing the dynamometer, which should be maintained for at least five seconds. Care will be taken to avoid Valsalva manoeuvres during this time. The average of the three attempts with a resting period of 15 seconds between attempts will be performed and the best value among them will also be recorded during the procedure ^38^.

### Sample size

Assuming a three-year cumulative event rate of 50% or more for the primary outcome (mortality) ^39^, it was estimated that a sample size of 1632 participants would provide 80% power to detect at least 20% relative risk reduction of the primary outcomes for each of the randomised comparisons.

### Randomisation and blinding 

Treatment allocation at each site will be assigned using an automated randomization process developed by an independent person who is not involved in the study. Computer-generated random numbers will be used for randomisation into four groups. Units of randomisation will be HF patients attending the outpatient department of the selected hospitals. Consenting participants will be allocated to one of the four groups (three intervention groups or one usual care group) in a 1:1:1:1 ratio using a central randomisation system. Randomisation will be stratified by site. The randomisation listing will be attached to the REDCap system for automatic online randomization upon completion of screening and collection of all baseline data. The trial is open-label, and patients and investigators will be aware of treatment allocation.

### Data management

All data collected from the patients and during the telephonic follow-up with each patient will be recorded in the study proforma. The data collection will be done using the REDCap application. All the participating centres will be given dedicated login credentials to enter the data. The data will be protected for privacy with the following standard precautions: We will assign a unique identification number to all participating centres and for each enrolled patient, so that they will be further identified only by that number; individual patient identification details will be collected and maintained only at the participating centres. The data will be entered by the research nurse under the supervision of the concerned cardiologist (investigator).

All the data fields in the REDCap application will be clearly defined and appropriate validation checks will be incorporated. For ease of operation, all study staff will have access to a procedural manual of operation. To ensure data validity and to identify discrepancies in data entry, the ‘edit check programs’ in REDCap will be used. Discrepancies or inconsistent data, missing data, range checks, and deviations will be identified. The data will be downloaded from the REDCap application and a data validation process will be run frequently to identify potential errors. All discrepancies will be resolved periodically. Ongoing quality control of data will be undertaken at regular intervals during data collection. Reviewing discrepancies, investigating the reason, and resolving them with documentary proof will be initiated in the data validation process.

The final data validation will be run after a proper quality check and assurance. If there are no issues, the datasets will be finalized in consultation with the study statistician. All data management activities will be completed prior to database lock. To ensure this, a pre-lock checklist will be used, and completion of all activities will be confirmed. Once the approval for locking is obtained from all investigators, the database will be locked, and the final data set will be extracted for statistical analysis.

A data management plan (DMP) will be created to understand who is responsible for creation, storage, and maintenance of the data and maintaining the privacy of the data collected. As per the DMP the authorized authority to view data will be the principal investigator and any other person as authorized by the principal investigator. The data will be saved in the inhouse server of SCTIMST.

### Statistical analysis

Baseline characteristics and follow-up measurements will be described using descriptive summary measures depending on the level and scale of measurements. Intention to treat analyses will be performed. The effects of intervention on the primary mortality endpoints will be estimated from unadjusted Cox proportional hazard models. We will combine mortality and hospitalisation for HF as a composite secondary outcome. To analyse the composite outcome, in participants with more than one outcome event during follow-up, survival time to the first relevant endpoint will be used in the analysis. As a general practice, all participants will be censored at their date of death or, for those still alive at the end of follow-up, or at the date of their last visit. Patients with an unknown vital status will be also right censored when they were last known to be alive. To calculate the relative risk reductions, we will use the formula based on the hazard ratio ([1–hazard ratio] ×100). The reciprocals of the absolute risk differences with their normally approximated 95% confidence intervals (CIs) will be calculated as the numbers needed to treat. For the continuous secondary outcomes variables, differences between randomised groups during follow-up will be estimated from linear mixed models or generalised estimating equations (GEE) as appropriate. In general, all p values will be calculated from two-tailed tests of statistical significance with a Type I error rate of 5%.

### Monitoring arrangements

The trial will be monitored by the data safety management board (DSMB). This will be an on-going activity from the time of initiation until trial close-out and will comply with the principles of Good Clinical Practice (GCP) and applicable regulatory requirements. The DSMB will monitor the overall conduct of the study to ensure the validity and integrity of the study findings, and will meet annually. The DSMB will comprise independent members with at least one statistician and one practicing cardiologist.

### Adverse events

All adverse events (AEs) that are directly observed and spontaneously reported by the research nurse and will be recorded in the CRF and reported to institutional ethics committee of the concerned hospital and coordinating centre. Death is considered as the serious adverse event in this study.

### Reporting and reproducibility

After completing the data analysis, the key findings will be published in academic journals related to cardiology, cardiac rehabilitation or behaviour sciences. A report will be submitted to the Indian Council of Medical Research (ICMR) for review and dissemination through their web portal and network. The data will be shared to the researchers, and clinicians involved in HF research after three years from completion of the study.

## Discussion

Cardiac rehabilitation is an important strategy for improving quality of life and survival of HF patients ^40, 41^. The PACT-HF trial will simultaneously examine the efficacy of two components of cardiac rehabilitation strategies ( *i.e.*, structured physical activity including exercise training, and cognitive behavioural therapy for depression, self-management) in improving survival and quality of life in HF patients from India. We will develop a culturally appropriate intervention model and implement it as a package of cardiac rehabilitation. It will ensure that the programme can be easily adopted as a formal cardiac rehabilitation strategy if it is found to be effective in improving survival and quality of life of HF patients in India. We will use resource-sensitive intervention tools in delivering the cardiac rehabilitation so that it is scalable in LMIC settings. Finally, we will use trained nurses in the delivery of the cardiac rehabilitation. This will also facilitate scale-up of similar intervention in wider health care delivery settings.

## Conclusions

The PACT-HF trial will answer whether physical activity-based training and cognitive behavioural therapy, which are part of the core components of cardiac rehabilitation, will be effective in independently improving survival and quality of life of HF patients in India. We will use culturally appropriate, resource-sensitive and context-specific strategies in the management of HF in Indian settings. The PACT-HF model could be adopted in similar health care settings in LMIC in delivering cardiac rehabilitation for HF patients. 

### Study status

The data collection for the study started in September 2021. The investigators are planning to complete the data collection by end of 2024.

## Data availability

### Underlying data

No data are associated with this article.

### Extended data

Figshare: A multicentric, 2 × 2 factorial, randomised, open-label trial to evaluate the clinical effectiveness of structured physical activity training and behavioural therapy versus usual care in heart failure patients: a protocol for the PACT-HF trial- study proforma and follow up form, https://doi.org/10.6084/m9.figshare.19112378.v2

This project contains the following extended data:

TIDier Check List: https://doi.org/10.6084/m9.figshare.19135010.v2

Figshare: Schedule for evaluation https://doi.org/10.6084/m9.figshare.19135010.v2

Figshare: Study design, https://doi.org/10.6084/m9.figshare.19137236.v1

Figshare: Exercise schedule, https://doi.org/10.6084/m9.figshare.19173821.v1

Figshare: Behaviour therapy- https://doi.org/10.6084/m9.figshare.19958654.v1

Data are available under the terms of the Creative Commons Attribution 4.0 International license (CC-BY 4.0).
