# Supplementary material for: A multicentric, 2 × 2 factorial, randomised, open-label trial to evaluate the clinical effectiveness of structured physical activity training and cognitive behavioural therapy versus usual care in heart failure patients: a protocol for the PACT-HF trial
Source: Wellcome Open Res. 2022 Aug 12;7:210. [Version 1] doi: 10.12688/wellcomeopenres.18047.1 (PMC9445562; doi:10.12688/wellcomeopenres.18047.1)
Supplement: Peer review history

# Peer review: Reviewer response for version 1

**DOI:** 10.21956/wellcomeopenres.20013.r52034

**Published:** 2022-09-05

**Authors:** Roopa Shivashankar

**Affiliations:** 1 Indian Council of Medical Research (ICMR), New Delhi, Delhi, India

The burden of cardiac failure (CF) is increasing in India. The CF patients in India are younger and have higher mortality compared to the Western cohort. Therefore, the cardiac rehabilitation program for CF adopted for Indian settings is relevant and timely. The project proposal is well written and clear. I also appreciate the factorial design of the study which provides better insight into the effectiveness of the individual components of the complex intervention. I have the following comments for the author’s consideration:

1. What does the usual care consist of? Is there standard care that is being practiced in all the trial centers or is there considerable variation across settings? If the current ‘usual care’ is inadequate, would the investigators consider providing enhanced/standard clinical and pharmacological care to patients in all arms?
2. The frequency of the intervention in the physical activity arm is unclear. How many face-to-face/virtual/telephonic interactions are expected between trainer and patient?
3. How many trainer nurses are in each center? Is the same nurse delivering both physical activity and cognitive behavioral therapy? If yes, how do the investigators prevent the contamination between arms?
4. In the discussion, the authors note that “We will develop a culturally appropriate intervention model and implement it as a package of cardiac rehabilitation. It will ensure that the programme can be easily adopted as a formal cardiac rehabilitation strategy if it is found to be effective in improving survival and quality of life of HF patients in India.” There are two issues in these statements. One, for the intervention to be easily adopted as a formal cardiac rehabilitation programme in India, there should be a cost analysis and a feasibility analysis (in terms of trainability and availability of nurses) of the intervention components. The investigators should consider adding these components to the trial. Two, the study is being done only in the state of Kerala, the success in Kerala may not be replicated in other parts of India where the health care settings and characteristics of the patient population are significantly different. This should be added as a limitation. 

Is the study design appropriate for the research question?

Yes

Is the rationale for, and objectives of, the study clearly described?

Yes

Are sufficient details of the methods provided to allow replication by others?

Yes

Are the datasets clearly presented in a useable and accessible format?

Not applicable

Reviewer Expertise:

Preventive cardiology; Non-communicable disease risk factors; hypertension; health systems research for NCDs

I confirm that I have read this submission and believe that I have an appropriate level of expertise to confirm that it is of an acceptable scientific standard.

# Peer review: Reviewer response for version 1

**DOI:** 10.21956/wellcomeopenres.20013.r52033

**Published:** 2022-08-19

**Authors:** Veena Nanjappa

**Affiliations:** 1 Department of Cardiology, Sri Jayadeva Institute of Cardiovascular Sciences and Research, Mysore, Karnataka, India

It is a well designed multicentric trial to study the time to mortality and composite of mortality and hospitalisation as outcome measures.

 The use of regional language and culturally acceptable exercise practices including yoga to assess the survival rate and quality of life is the need of the hour in the Indian scenario.

 The main aspect of the study will be to ensure adequate training and follow up of the training and achieve standardised delivery of care - physical and behavioural training at multiple centres.

 Secondly, the self help manuals can be made into audio clips for those who are illiterate.

Is the study design appropriate for the research question?

Yes

Is the rationale for, and objectives of, the study clearly described?

Yes

Are sufficient details of the methods provided to allow replication by others?

Yes

Are the datasets clearly presented in a useable and accessible format?

Yes

Reviewer Expertise:

heart failure, interventional Cardiology

I confirm that I have read this submission and believe that I have an appropriate level of expertise to confirm that it is of an acceptable scientific standard.
